# Cultural practices, healthcare-seeking behaviors, and wildlife interface: Zoonotic disease risks among the Phu Thai Ethnic Group in Thailand

**DOI:** 10.14202/vetworld.2025.624-635

**Published:** 2025-03-18

**Authors:** Nisachon Bubpa, Kanokwan Suwannarong, Kannika Thammasutti, Thanomsin Ponlap, Worakamon Thongkan, Paisit Boonyakawee, Phitsanuruk Kanthawee, Kangsadal Suwannarong, Withaya Chanchai

**Affiliations:** 1 Department of Family and Community Nursing, Faculty of Nursing, Khon Kaen University, Khon Kaen, Thailand; 2 Center of Excellence for Emerging and Re-emerging Infectious Diseases in Animals, Chulalongkorn University, Bangkok, Thailand; 3 SUPA71 Co., Ltd, Bangkok, Thailand; 4 Sirindhorn College of Public Health Trang, Praboromarajchanok Institute, Ministry of Public Health, Trang, Thailand; 5 Unit for Area-based Research and Innovation in Cross-Border Health Care, Department of Public Health, School of Health Science, Mae Fah Luang University, Chaing Rai, Thailand; 6 The Office of Disease Prevention and Control 7 Khon Kaen, Khon Kaen, Thailand; 7 Department of Public Health, Major in Occupational Health and Safety Program, Faculty of Medicine, Siam University, Bangkok, Thailand

**Keywords:** coronavirus disease 2019, emerging infectious diseases, healthcare-seeking behaviors, Mukdahan, One Health, Phu Thai, Thailand, wildlife interface, zoonotic diseases

## Abstract

**Background and Aim:**

Emerging infectious diseases, with 75% originating from zoonotic sources, highlight the interconnectedness of human, animal, and environmental health. The coronavirus disease 2019 (COVID-19) pandemic underscored the importance of the One Health (OH) approach, especially in rural and ethnic communities where cultural practices and wildlife interactions may amplify zoonotic disease risks. This study determined the healthcare-seeking behaviors and wildlife interface of the Phu Thai ethnic group in Mukdahan Province, Thailand, to understand their cultural practices, zoonotic disease risks, and pandemic-related adaptations.

**Materials and Methods:**

From June to July 2023, a qualitative study was conducted in three villages of Nong Sung District, Mukdahan Province. Data collection included 3 focus group discussions (16 respondents), 6 in-depth interviews, and 5 key informant interviews, with a total of 27 respondents consisting of community members, leaders, and government officials. Thematic analysis was performed to explore cultural traditions, wildlife interactions, healthcare practices, and perceptions of COVID-19.

**Results:**

The Phu Thai people maintain a deep connection to cultural traditions, including ancestral rituals and wildlife use for food and ceremonies. While traditional practices such as consuming raw wildlife persist, the COVID-19 pandemic has significantly influenced their attitudes, leading to increased caution and community-driven preventive measures. Limited knowledge about zoonotic diseases and unsafe practices, such as handling wildlife without protection, were identified as risk factors. Accessibility to healthcare services was moderate, with language barriers and resource constraints posing challenges. However, the community demonstrated resilience by adopting local initiatives such as mask-making and remote traditional healing.

**Conclusion:**

This study highlights the complex interplay between culture, healthcare access, and zoonotic risks in the Phu Thai community. Enhancing culturally sensitive health education, promoting safe wildlife interaction practices, and leveraging the OH framework can reduce zoonotic disease risks while respecting traditional practices. The findings suggest that key stakeholders, such as community members, leaders, traditional healers, public health officers, local authorities, and relevant stakeholders, should be informed to gather their feedback and support in improving policies and regulations related to wildlife contact and practices. These efforts are expected to contribute to sustainable health outcomes and align with Sustainable Development Goals (SDGs) 3 (health and well-being) and SDG 12 (responsible consumption and production).

## INTRODUCTION

Emerging infectious diseases (EIDs) are infections that spread rapidly across populations, with approximately 75% originating from zoonotic sources and circulating among animal hosts [1]. Notable examples of EIDs include Ebola, Middle East respiratory syndrome (MERS), avian influenza (H5N1), swine influenza (H1N1), and severe acute respiratory syndrome (SARS) [2, 3]. The coronavirus disease 2019 (COVID-19) pandemic underscored the critical need for healthcare systems to enhance their preparedness for future pandemics [4]. Research by Racciatti *et al*. [5] demonstrate that the close interactions between humans, the environment, and animals, including wildlife, may have played a significant role in the emergence of the pandemic. This situation has emphasized the relevance of the One Health (OH) approach, which addresses health challenges across human, animal, and environmental domains [6]. The OH concept highlights the interconnectedness of these sectors and acknowledges the vital contribution of animal health to public health [7]. A systematic review of SARS, MERS, and COVID-19 outbreaks has documented the evolution of the OH approach and identified essential measures for prevention, response, and control [8].

Cultural beliefs, values, and norms substantially influence health perceptions, illness management, and public health strategies during pandemics [9]. In addition, culture shapes dietary practices, often hindering healthy eating due to traditional beliefs and social influences [10, 11]. Healthcare-seeking behaviors differ among ethnic groups, shaped by cultural, social, economic, and historical contexts. Many communities rely on traditional healing practices deeply embedded in their heritage [12]. The Phu Thai ethnic group, an indigenous community in northeastern Thailand, Laos, and Vietnam, exemplifies this cultural heritage [13]. In Thailand, the Phu Thai primarily reside in provinces such as Kalasin, Nakhon Phanom, Sakon Nakhon, Mukdahan, Amnat Charoen, and Yasothon [14]. In Nong Sung District, Mukdahan Province, the Phu Thai community upholds rich cultural traditions, including traditional healthcare practices and herbal remedies that are integral to their community health management [15]. Their practices often involve ceremonies led by shamans or traditional healers to ward off evil spirits or promote health [13, 16]. For healthcare providers, understanding and respecting these cultural beliefs are crucial to delivering culturally competent care, particularly during crises such as the COVID-19 pandemic [17].

At present, limited information exists regarding the interactions between the Phu Thai ethnic group and wild animals, as well as their healthcare-seeking behaviors during pandemics. Therefore, this study aimed to explore the healthcare-seeking behaviors and wildlife interface practices of the Phu Thai ethnic group in Nong Sung District, Mukdahan Province, Thailand. The research specifically examined respondents’ cultural traditions, perceptions of zoonotic disease risks, and how the COVID-19 pandemic influenced their attitudes and behaviors. The study’s findings are expected to contribute to communication interventions that enhance health literacy, promote safe wildlife interactions, and mitigate zoonotic disease risks while advancing Sustainable Development Goals (SDG 3 and SDG 12) [18, 19].

## MATERIALS AND METHODS

### Ethical approval and informed consent

Ethical approval for this study was obtained from the Research Ethics Review Committee for Research Involving Human Research Respondents, Health Sciences Group, Chulalongkorn University (Ref No. 150.1/64). The study adhered to ethical principles, including respect for each person, beneficence by expecting benefits for the community, and justice by ensuring equal rights and protection for all respondents. This study’s implementation followed the guidelines of the Declaration of Helsinki. The study respondents were informed about the study objectives, procedures, and their rights to refuse or withdraw at any time without any impact on their ability to obtain public services or activities. The respondents signed written informed consent forms before participating in the data collection activities. The collected information was anonymized, which made it impossible to trace their identification.

### Study period, design, and location

**Figure 1:**
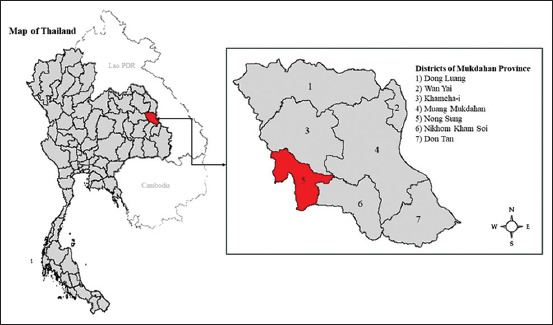
Map of Nong Sung District in Mukdahan Province [20].

This qualitative study was conducted and implemented during June and July 2023. We employed in-depth interviews (IDIs), key informant interviews (KIIs), and focus group discussions (FGDs) to gather information on wildlife’s contact characteristics, perceptions, and healthcare-seeking behaviors regarding COVID-19, including their risks from wildlife contacts among the Phu Thai in three communities in Nong Sung District of Mukdahan Province (Figure 1) [20]. Village No. 1 has a population of 619, Village No. 2 has a population of 450, and Village No. 3 has a population of 281, respectively [20]. These areas were selected because they had the highest numbers of Phu Thai [20]. Village No. 1 is a Phu Thai cultural village with the most extended community history [21]. The study sites are located in the northeastern region of Thailand, which connects to Laos’ Savannakhet Province through the Mekong River bridge. It borders the Phu Phan Mountain range and is known for its diverse indigenous ethnic groups. These areas are rich in natural resources, including forests, wildlife, and mountains, which support their livelihoods.

### Study respondents

This qualitative study followed qualitative research method guidelines for determining an appropriate sample size [22–24]. Guest *et al*. [25] suggested that a sample size of 12–20 respondents is generally sufficient to achieve data saturation in qualitative studies. This study achieved saturation when no new themes emerged after analyzing the responses from 27 respondents. In addition, a purposive sampling method was employed for this study in a cluster of Phu Thai ethnic groups [26] using a purposive selection method, which allowed us to obtain diverse and comprehensive information among the target population. Phu Thai villagers from the chosen communities were invited to participate based on referrals from local authorities and community members. They were born and raised in these villages and lived in the communities for at least 12 months before the study.

### Study tools

Discussion guides for the IDI, KII, and FGD were adapted from a previous study by Boonyakawee *et al*. [27]. The gathered information included geography characteristics, occupations, social and economic characteristics, culture, and beliefs; area problems, such as main health problems, solutions, and access to healthcare or health-related services; activities related to wildlife, including knowledge, attitudes toward self-protection and community, beliefs, and consumption; and knowledge, attitudes, self-protection, community protection, healthcare-seeking behaviors, and perceptions toward COVID-19.

### Data collection and analysis

Data collection was conducted by the trained researchers using IDI, KII, and FGD guides, with written consent obtained from all respondents before participation. The interviews and discussions were audio recorded and transcribed verbatim into transcriptions from the local northeastern dialect to the central Thai language. All transcriptions were reviewed by facilitators and note-takers for accuracy before analysis. We used thematic analysis to identify themes and coding data. The major analysis themes included: (1) Profiles of the study respondents, (2) cultures and beliefs among the Phu Thai, (3) social structures, (4) health issues of the Phu Thai, (5) wildlife interface characteristics, (6) reasons for consuming wild animals, (7) diseases caused by wild animals, (8) taboos of the Phu Thai toward wildlife, (9) knowledge, attitudes, practices, and beliefs of the Phu Thai people toward healthcare and COVID-19, (10) perceptions and impacts of the COVID-19 pandemic, and (11) accessibility to health services. These themes were developed through coding, analyzing, and managing data using NVivo software, version 14 (https://lumivero.com/products/nvivo/) in the qualitative data analysis (QDA) process and were validated by a team of experts and researchers for accuracy [28]. To enhance data reliability and validity, we employed triangulation analysis [28] by cross-verifying findings from various data collection methods (IDIs, KIIs, and FGDs), having multiple researchers and experts review the data and results to reduce bias and misinterpretation of the gathered information. In addition, we shared the brief findings with the study respondents, community members, and stakeholders to validate the results and gather feedback before further analysis. This validation process improved the findings’ accuracy, cultural sensitivity, and trustworthiness.

## RESULTS

### Profiles of the respondents

The study included 27 respondents, comprising 14 males (51.85%) and 13 females (48.15%), all of whom were Phu Thai ethnic people and practicing Buddhists. Their occupations varied, with the majority being farmers (18 individuals, 66.67%), followed by retired civil servants (3 individuals, 11.11%), shopkeepers (2 individuals, 7.41%), community leaders (2 individuals, 7.41%), a resort manager (1 individual, 3.70%), and a government officer (1 individual, 3.70%). Data collection involved 16 respondents in 3 FGDs, 6 IDIs, and 5 KIIs. The mean age of the respondents was 52.15 years (range: 35–63 years, standard deviation = 7.62) (Table 1).

**Table 1:** Sociodemographic information about the respondents.

Description	Frequency	Percentage
Sex		
Male	14	51.85
Female	13	48.15
Age (years old)		
30–39	2	7.41
40–49	9	33.33
50–59	10	37.04
60–69	6	22.22
Mean=52.15, SD=7.62, Minimum=35, Maximum=63		
Ethnicity		
Phu Thai	27	100
Religion		
Buddhism	27	100
Occupations		
Farmer	18	66.67
Retired civil servant	3	11.11
Shopkeeper	2	7.41
Community leaders	2	7.41
Resort manager	1	3.70
Government officer	1	3.70
Total	27	100.00

SD=Standard deviation

### The cultures and beliefs of the Phu Thai

The term “Phu Thai” refers to the language of the largest Tai-Lao ethnic group in northeastern Thailand, which is separated from the Mekong River Basin’s Phu Thai group by the Mekong River and the Phu Phan Mountains. The Phu Thai in these study locations maintains traditional practices and cultural heritage. The Phu Thai mainly follow Theravada Buddhism and maintain beliefs in karma and ancestral spirits, living in harmony with nature, while a minority practice Christianity. The community values its culture, traditions, and ancestral respect, known as “San Jao Pu Ta,” which fosters unity.

The Phu Thai ethnic group is known for maintaining its unique cultural identity and traditions. They engage in farming, including rice, rubber, and seed grass cultivation, with some focus on fruit tree plantations such as mangoes and bananas. Livestock farming includes cows, pigs, and goats. The community also has enterprises producing mulberry leaf tea and traditional fabrics. A general labor group is also involved in construction, trading, and seed collection. Some young people study or work in Bangkok, which results that most people in the area being children and elderly, with the population growing due to family expansion and migration for marriage. Most Phu Thai earn money from seasonal agriculture. They raise animals and grow vegetables, using water from ponds.

The Phu Thai follow Theravada Buddhism and practice ancestral spirit worship, using wild animals for ceremonial purposes. These traditions, distinct from Isan (northeastern) culture, shape their lives, including healthcare and trade. Their traditions include the Boon Pha Wuet Ceremony, an annual event that takes place between villages every 3 years. Before the ceremony, they prepared ceremonial tools called “Kreung Pan” to ward off rainstorms. The water was drawn from a stream for worship, and a sacred priest blessed it for prosperity. The ceremony involves the “Leplern Phii” or “Ao Hoop Ao Hoy” play, burying figures symbolizing well-being, health, and fertility. They also practice Spirit Mediumship (Mor Yaow), in which a healer predicts agricultural conditions through dance, with forest spirits protecting the community. The creatures dressed as forest spirits must continue for 3 years to prosper. They also trusted traditional indigenous medicine and herbal remedies.

In addition, the Phu Thai tradition of venerating the ancestral spirit “Pu Ta” (holy place) has been practiced since ancient times and is believed to reside among them for protection. The Pu Ta shrine, which was designed like a traditional Thai house, was located near a stream. Phu Thai made offerings to Pu Ta for various reasons, with a grand ceremony every 3 years. They also believed in traditional healers and herbal medicine and used black magic for treatment when modern methods failed, or the cause of illness was unknown. The Mor Yaow ceremony was used to identify unknown illnesses caused by headaches or diarrhea.

### Social structures

The Phu Thai ethnic group highly valued their paternal and maternal grandparents, known as Phor Lam and Mae Lam, as they were considered the foundation of the Phu Thai cultural heritage. These Phor Lam and Mae Lam grandparents played crucial roles in traditional marriage customs, symbolizing wisdom in building strong family bonds and harmonious societies. The Phor Lam and Mae Lam culture is considered a source of family ties and cultural capital that has existed since previous periods and has lasted to the present day. When a young couple decided to marry, the man sought guidance from individuals known for exemplary household management skills, including advice on health care.

### Health issues of the Phu Thai people

They reported the following health issues: Livestock diseases like lumpy skin disease, addressed through livestock vaccination. Residents also faced non-communicable diseases such as diabetes, hypertension, liver disease, and others, detected more quickly with modern medical tools. Musculoskeletal diseases and fatigue are common in seniors. Communicable diseases include influenza, dengue fever, and melioidosis, with isolated cases of HIV and COVID-19.

### Wildlife interface characteristics

#### Wildlife interface and consumption practices

In the past, the Phu Thai relied on hunting and gathering in forests, adjusting to seasonal availability. With no restrictions or limited amenities, they harvested wildlife such as wild boar, deer, porcupine, and others from abundant areas like Jok Koe Mountain. They have shifted to farming or purchasing wildlife because of declining populations and wildlife protection laws that prohibit hunting in certain areas. Commonly raised animals include rabbits, rats, wild boars, and jungle fowls. In daily life, they buy these animals to cook at special events, such as parties, ceremonies, or when friends visit. However, if they knew that hunters had caught wildlife animals, they would buy and eat them, such as bats, rabbits, wild rats, and chipmunks. The ways of eating were still the same as in the past, such as with raw blood and meat, because the taste was good. During the ancestor respect seasons, some of them decided to cook wild animals in ancestral styles.

*“This blood must be collected. The spicy raw rabbit salad is so tasty, especially its belly.”*

*(A farmer in a FGD, MH_FGD_001)*

The Phu Thai frequently prepared wildlife dishes in both raw and cooked forms. The raw meat was consumed, and it was assumed that it was free of pathogens. Raw dishes commonly include cicadas, deer, rabbits, and wild boar and are used in dishes like Larb (spicy meat salad) and Som Tam (spicy papaya salad), often paired with mango or Jaeo (a type of chili paste). For cooked dishes, birds, rats, squirrels, porcupines, and monitor lizards were popular choices and were used in dishes such as local curry (Kaeng Om), meat salad (Larb), stir-fries, soups, and spicy stir-fried snake dishes. Monitor lizards were also cooked with eggplant or Thai basil.

*“Cicadas can be eaten raw because they are found in the sky and are free from germs.”*

(*A farmer in a FGD, MH_FGD_003*)

**Figure 2:**
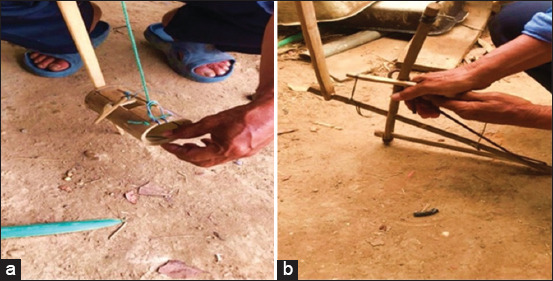
(a and b) Huntsman demonstrating the use of various bamboo traps.

Wildlife was mainly sourced from traders in Ngiw and Wang Nong villages, who often obtained it from Laos. Some also purchase wildlife from acquaintances or at the village markets every Wednesday. Wildlife is typically hunted using traps such as snare traps, loop and shake traps (to lure rodents), and bamboo traps, often set in agricultural areas (Figure 2). Some Phu Thai also raised wild animals for food or sale, including squirrels, which were sold at a rate of 300 Thai Baht (around 9 USD) per kilogram. However, most wildlife was not sold openly.

*“Most of them* (*wildlife*) *were not sold openly. They were sold secretly.”*

*(A farmer, MH_IDI_F_004)*

Traditional hunting, using local traps, was once a significant tradition for the Phu Thai, but it declined because of the country’s firearm and plant protection laws. The police would confiscate firearms if individuals carried them. The hunters had to hide it in a safe place. In the past, hunters, known as “Nay Pran,” would go in large groups carrying guns to hunt deer and wild boar, often tracking animals at night using footprints and excrement. Wild boar hunting involved surrounding the animals, with “Tai Don” acting as the decoy. Nowadays, some Phu Thai still hunt seasonally, with specific practices such as burning and cooking fresh bamboo shoots in November when mother boars are pregnant and using the boar’s belly to make chili paste.

*“Nowadays, we can’t carry firearms because the police will confiscate them.”*

*(A farmer, MH_KII_M_004)*

During the rainy season, traps were set in banyan and ironwood trees to catch cicada larvae and female moths during the egg-laying period. Female moths were harvested as caterpillars before they became pupae and then roasted and seasoned. Cicadas were found in cold mountainous areas in winter, emitting sounds similar to geckos. Hunters used battery-operated headlights to locate the cicadas, which were then cooked in a bitter curry. Our interviews revealed that men were primarily responsible for hunting wild animals, while women were responsible for cooking. Both genders were involved in the processing of the wild boar. The first method involved immediate butchering in the forest, which involved burning off the hair, cleaning the carcass, and removing unwanted parts, such as the intestines, legs, and blood, which were collected in a bag. The meat was quickly cut and distributed among the hunters. The liver was cooked and served with a dipping sauce made from water and liver, seasoned with roasted rice powder, chili, and fish sauce. In the second method, the wild boars were large and required the assistance of 4–5 friends to butcher the animals in the rice fields. The meat was distributed to acquaintances and neighbors rather than being sold. Women processed porcupines, deer, wild pigs, and rats by burning off their fur, skinning and gutting them, and then cleaning them. The meat was finely chopped for spicy stir-fry, lab, and stir-fry dishes. No protective covers or gloves were used. The inedible portions of the python skin were removed after burning and cleaning. The remaining portion was boiled until tender, seasoned, and then consumed as a soup. This dish is widely available for purchase and consumption.

*“*(*We*) *Don’t wear gloves because they think they* (*wildlife*) *are no diseases.”*

*(A farmer, MH_KII_M_003)*

### Reasons for consuming wild animals

The Phu Thai consumed wild animals because of tradition, followed by the previous generations, poverty, availability, taste, and influence, with some eating them for protein. Others avoided them to protect wildlife because of cost, health concerns, and alternatives. Wild animals were used in ceremonies and traditional dishes, reflecting traditional practices. The Phu Thai also practiced “eating the year,” consuming wild animals seasonally, such as the “Eung-ang” bird or red ant eggs. However, some refused to consume wild meat because of its unpleasant odor.

### Diseases caused by wild animals

Some respondents reported that the Phu Thai people were aware of diseases caused by eating raw wild animals. They prevented these diseases by thoroughly cooking food. Some respondents reported that the Phu Thai people were aware of several diseases linked to wild animals and had their own beliefs about these diseases. They thought deafness (*Streptococcus suis*) originated from eating raw or undercooked pork. Some respondents mentioned that diarrhea was caused by eating rotten or poorly cleaned wild animal meat. They explained that leptospirosis could occur after contact with water contaminated with animal urine, especially in flooded rice fields. Some respondents reported avian influenza from handling or eating infected poultry. They also believed that gout could result from eating wild animals, such as rats and porcupines, which they thought were high in uric acid. Despite their concerns about diseases caused by consuming wildlife, they continue to seek them out for consumption.

*“We know there are diseases, but the villagers still kill and eat the animals. But for me, it tastes delicious.”*

(*A farmer in a FGD, MH_FGD_001*)

### Taboos of the Phu Thai toward wildlife

**Figure 3:**
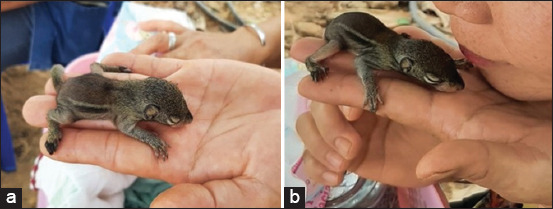
(a and b) Community members collect fallen chipmunks from trees and raise them as pets out of compassion for the young animals.

The hunting taboos surrounding the community were classified into two distinct categories: (1) The community has implemented regulations, such as prohibiting fishing in the village pond, except during approved periods. Violators will be warned, and insecticide use in rice fields is not allowed and (2) the forestry department regulations strictly forbid hunting in three designated mountain areas: Teuk Khaokor, Phu Jokkoe, and Phu Sitthasarn. Violators of these regulations may face fines ranging from 200 to 6,000 Thai Baht (around 6–180 USD), as well as potential disciplinary measures and community service. Approximately 80% of the locals complied, with the remaining 20% hunting illegally in the mountains. Furthermore, it appears that some villagers and children engaged in the practice of catching and keeping wild animals as pets (Figure 3).

*“They say if you catch a bird, you will be in jail for a year; that’s what they say.”*

(*A farmer, MH_KII_M_005*)

Some respondents mentioned that those who had just given birth should not eat rabbits or wild animals; otherwise, this would cause severe illness or death for the mother. Traditions prohibit specific groups from eating certain meats to prevent childbirth complications or mouth ulcers. Some Phu Thai community members hunt or eat certain animals because of their spiritual beliefs. For example, they avoid snakes and tigers because they are believed to have strong spirits or bring bad luck. Owls and crows are linked to death or bad news, whereas monkeys are considered unique due to their similarity to humans. These beliefs help them maintain spiritual balance and avoid adverse events.

*“There was a prohibition for women who had just given birth to consume wild animals, for example, rabbits. If they have it, women might have convulsions or die.”*

(*A farmer in a FGD, MH_FGD_001*)

*“Some animals, like snakes and tigers, have strong spirits and can bring bad luck, while owls and crows are signs of death. We avoid them to stay safe and keep the spirits happy.”*

(*A farmer in a FGD, MH_FGD_001*)

The key findings of wildlife interface characteristics among the Phu Thai ethnic group are summarized in Table 2.

**Table 2:** Key findings on wildlife interface characteristics among the Phu Thai ethnic group.

Wildlife interface characteristics	Categories	Key findings
Wildlife interface and consumption practices	Hunting Practices	• The Phu Thai traditionally relied on hunting activities, but these practices have been reduced due to wildlife protection laws and regulations, including the decline in wild animal populations.
		• Hunters used snare traps, loop traps, shake traps, and firearms and were hunted in groups. Currently, wildlife protection laws and local regulations enforce the use of firearms.
		• The Phu Thai hunted wild animals on some occasions, such as hunting wild boars during bamboo shoot harvests.
	Sources of wild animals	• Due to restrictions on hunting wild animals under wildlife protection laws and local regulations, most community members rarely hunt and usually buy wild animals from local markets and traders within their communities.
		• Some Phu Thai raised wild animals, such as squirrels, rabbits, and jungle fowl, for food or sale.
	Consumption of wild animals	• The Phu Thai consume wild animals during ancestral ceremonies and traditional rituals, often linking them to spiritual beliefs.
		• The raw dishes of Phu Thai include deer, rabbits, and wild boar, which are often prepared in spicy meat salads (Larb).
		• Popular cooked wild animals included birds, rats, squirrels, porcupines, and monitor lizards, which were prepared in soups, stir-fries, and curries.
		• Some wild animals, such as cicadas and red ant eggs, are consumed seasonally, depending on their availability.
	Food preparation practices	• Men typically hunt wild animals, while women are responsible for cleaning, processing, and cooking meat.
		• Women skinned, gutted, and chopped wild animals, such as rabbits and wild boar, without using protective gear or hygiene practices.
Reasons for Wild Animal Consumption among Phu Thai	Tradition and culture	• The Phu Thai consumed wild animals as part of long-standing traditions and cultural practices passed down from previous generations.
		• The Phu Thai used wild animals in important ceremonies.
	Taste and preference	• Many Phu Thai people prefer the taste of wild animal meat, which is often considered more flavorful than that of farmed meat.
		• Some of them consumed wild animals to obtain nutrients and protein.
	Seasonal consumption	• Some Phu Thai consume wild animals, such as cicadas and red ant eggs, as seasonal foods during specific seasons.
Diseases reported to occur by casing by contacting or consuming wild animals	Deafness (*Streptococcus suis*)	• Deafness (*Streptococcus suis*) is caused by eating raw or undercooked pork.
	Diarrhea	• Diarrhea is caused by the consumption of rotten or poorly cleaned wild animal meat.
	Leptospirosis	• Leptospirosis is reportedly caused by contact with water contaminated with animal urine, often in flooded rice fields.
	Avian influenza	• Avian influenza is caused by handling or consuming infected poultry.
	Gout	• Gouts were reported to be caused by the consumption of wild animals such as rats and porcupines, which are believed to be high in uric acid.
Taboos and hunting restrictions	Prohibited hunting areas: hunting areas that do not permit hunting	• According to government regulations, hunting is strictly forbidden in the three designated mountain areas (Teuk Khaokor, Phu Jokkoe, and Phu Sitthasarn Mountain areas).
	Taboos	• Women who have just given birth are forbidden from consuming wild animals such as rabbits or specific meat due to the belief that they can cause sickness.
		• To respect traditional spiritual beliefs, some community members avoided hunting or consuming animals, such as snakes, owls, tigers, and monkeys.

### Knowledge, attitudes, practices, and beliefs of the Phu Thai people toward healthcare and COVID-19

The Phu Thai attribute unexplained illnesses to displeased spirits, including ancestors, seeking treatment from traditional healers like “Mor Yaow” (Phu-Thai shamans) or “Mor Pao” (Magic speller). These folk healers used rituals involving prayers, offerings, and ceremonies to appease spirits and conducted merit-making ceremonies and charitable acts for recovery. For severe illnesses that persisted without improvement, the Yaow ritual was used to aid recovery from unknown illnesses. Magic spellers usually employ magic spelling with herbs or tree bark for various treatments, often combining traditional and modern practices for pain relief and treating bone-related issues, snake bites, and diseases.

*“If modern medication fails, we consult a Mor Yaow because they offer various treatments.”*

(*A farmer in a FGD, MH_FGD_003*)

*“Mor Pao’s treatments are quick and effective, unlike Mor Yaow, which involves longer ceremonies and offerings.”*

(*A farmer, MH_KII_M_003*)

Choosing a traditional healer was based on faith and reputation, considering track record, experience, and community recommendations. At present, 30% of the population prefer traditional healing, a shift from 100% in the past. Some relied on healers, such as “Mor Phi” (Shaman), when modern treatments failed. The treatment cost varies, starting at 1,000–2,000 Thai Baht (29.60–59.20 USD) for rituals by “Mor Yaow” and 9-200 Thai Baht (0.27–5.92 USD) for “Kha Khru (fee).” Ancestral and forest spirits were integral to healthcare beliefs, involving traditional healers like “Mor Yaow,” who acted as intermediaries, using rituals and incantations to treat illnesses and dangers, showcasing the group’s adaptability. During the COVID-19 lockdown, residents of Bangkok could not travel home. Some people sought Mor Yaow’s remote traditional healing services over the phone, while others made virtual direct calls through LINE communication platform. They believed that this would improve their health. Some Phu Thai had enough money, so they rented a vehicle for Mor Yaow to perform religious activities in Bangkok.

*“During COVID-19, some* (*Phu Thai*) *sought remote traditional healing services when unable to travel. They consulted healers over the phone, believing they could improve their* (*health*) *condition. Some hire traditional healers to perform rituals in Bangkok.”*

(*A retired civil servant in a FGD, MH_FGD_002*)

### Perceptions and impacts of the COVID-19 pandemic

The Phu Thai primarily relied on village leaders, hospital staff, and village health volunteers (VHVs) for information, supplemented by television (TV), phone, and online sources such as Facebook, LINE, and YouTube. They trusted information from village leaders, hospital staff, and VHVs the most, followed by TV, phone, face-to-face interactions, and any updated news dissemination by village leaders through a loudspeaker every morning. Village leaders were trusted for their knowledge and official status, while hospital staff and VHVs were valued for their ability to conduct tests and provide vetted information. TV and phone information was moderately trusted but may require verification, while face-to-face communication was least trusted due to potential errors. Recommendations included verifying information from TV and phone and citing news sources, especially regarding COVID-19. Villagers have adapted their lifestyles and prepared for surveillance during festivals due to COVID-19, with some unsure about its origin but rejecting the idea that eating bats caused the disease. They believe that it might have leaked from a laboratory in Wuhan, China, as reported on TV, due to the consumption of exotic animals. However, they did not think that the consumption of exotic animals in their community could be contaminated because these animals live naturally without illness.

*“I think in our village, people rely on natural resources for their livelihood. They eat fruits from the forest, and our ancestors consumed them without any illness.”*

(*A community leader, MH_KII_F_001*)

During the initial COVID-19 outbreak, the community collaborated with a military mobile team to prevent the virus’s spread. They prepared by growing vegetables and organizing mask-making training sessions using funds from the village’s health promotion project. When the lockdown was implemented, village leaders ensured that every household wore masks. In another community, 9 cases were reported, and deaths were mainly due to underlying health conditions. Village leaders suspended traditional ceremonies, restricted travel after 10:00 P.M., and emphasized mask-wearing. The monks stayed in a temple and did not go out for alms. The villagers had to bring food to offer at the temples. Hospitals were set up in waiting areas, and field hospitals and child development centers served as temporary quarantine facilities. Village leaders and public health officers supported the community by providing canned fish, dry food, and rice to the patients, and a group of LINE communication platform was created to report and monitor the situation.

*“During that time, the monks stayed in* (*temple*) *and didn’t go out for alms. They* (*monks*) *received food offerings and saved them.”*

(*A retired civil servant in a FGD, MH_FGD_002*)

As the COVID-19 situation improved, infected Phu Thai residents chose self-treatment, using herbal medicine like “Far Talai Jone” (*Andrographis paniculata*) pills and isolating for 2–3 days or until negative antigen test results. They remained vigilant, prioritizing news updates and surveillance, actively seeking high-risk groups, and monitoring the self-treatment of infected individuals through VHVs reporting to hospitals. Most residents received 2-3 vaccine doses following the guidelines of the Local Administration Organization and Public Health officers. Some reserved vaccines through the Department of Provincial Administration and the Ministry of Interior’s application for Digital life and Secured identity, while others chose AstraZeneca and Moderna. They also boiled herbal medicines such as lemongrass, kaffir lime leaves, and galangal.

The key findings of knowledge, attitudes, practices, and beliefs of the Phu Thai ethnic group regarding healthcare services and COVID-19 are summarized and presented in Table 3.

**Table 3:** Key findings on knowledge, attitudes, practices, and beliefs among the Phu Thai ethnic group regarding healthcare services and COVID-19.

Knowledge, attitudes, practices, and beliefs of the Phu Thai People toward Healthcare and COVID-19	Categories	Key findings
Beliefs and practices of the Phu Thai people regarding healthcare services and COVID-19	Traditional healing practices	• The Phu Thai believe that spirits, including unhappy ancestors, cause illnesses.
		• The Phu Thai sought help from traditional healers such as “Mor Yaow” (Phu Thai shamans) or “Mor Pao” (magic speller), who performed rituals, prayers, and offerings.
	Selection of healers	• The selection of a healer is based on faith, reputation, and community recommendations.
	Treatment costs	• Rituals cost around 1,000–2,000 Thai Baht (29.60–59.20 USD), while traditional fees known as Kha Khru cost around 9–200 Thai Baht (0.27–5.92 USD).
	Remote healing methods during COVID-19	• During the COVID-19 lockdown, Phu Thai people living in Bangkok sought remote healing services from “Mor Yaow”. They contacted Mor Yaow through phone and video calls through online platforms.
		• Some Phu Thai people hired Mor Yaow to travel to Thailand and perform Yaow rituals in person, which included prayers, chants, offerings, and ceremonies to appease spirits and treat illnesses.
Perceptions and impacts of the COVID-19 Pandemic	Sources of information	• The Phu Thai relied on village leaders, hospital staff, and VHVs for reliable information.
		• They also use TV, phones, Facebook, LINE, and YouTube to stay updated.
	Information trust levels	• The Phu Thai trusted village leaders, hospital staff, and VHVs most because of their knowledge and authority.
		• They moderately trusted TV and phone updates.
		• They least trusted face-to-face communication because of potential errors.
	COVID-19 prevention	• The Phu Thai learned how to make masks through training programs. Village leaders ensured that every household wore masks and stopped ceremonies during the COVID-19 lockdown.
		• Local government and health staff set up temporary quarantine facilities in hospitals and child development centers and provided patients with food such as rice and canned fish.
		• The Phu Thai people also used Far Talai Jone (*Andrographis paniculata*) pills and herbal remedies such as boiled lemongrass, kaffir lime leaves, and galangal.
	Vaccination and monitoring	• Most Phu Thai community members received 2–3 doses of COVID-19 vaccines, following public health guidelines.
		• VHVs screened and reported COVID-19 cases to health staff using communication platforms like LINE.

COVID-19=Coronavirus disease 2019, VHVs=Village health volunteers, TV=Television, USD=United States Dollar

### Accessibility of health services

Access to Phu Thai treatment was generally fair, with a line system in place. However, disparities in treatment rights under the healthcare social security scheme resulted in longer wait times and limited medication options. Some opt for private clinics for immediate treatment. The Phu Thai language is not spoken by certain hospital staff members, particularly young medical doctors who originate from other provinces. Delays in understanding were primarily caused by a lack of interaction with the staff, particularly the seniors. To facilitate communication and translation, young relatives must accompany them. The majority of Phu Thai residents independently used hospital services and sought advice from village leaders and health volunteers regarding disease screening. When they were unable to visit the hospital, some individuals used emergency services, or a hotline call from municipal council members.

*“Communication challenges arise for senior Phu Thai villagers when visiting doctors. They often bring grandchildren or caregivers to help, and doctors allow these helpers to listen for better understanding.”*

(*A community leader, MH_KII_F_001*)

## DISCUSSION

The Phu Thai community has preserved its ancestral traditions and cultural identity over generations, honoring their ancestors through ceremonies and maintaining their distinctive language. Although traditional hunting and gathering practices have diminished over time, the community has remained closely bonded, particularly during cultural ceremonies. Traditional healing practices, such as “Mor Yaow,” are still valued, though their prominence is gradually declining due to the increasing adoption of modern medicine. Saensila *et al*. [29] observed that traditional healers acted as intermediaries between patients and spirits, particularly when illnesses were believed to result from spiritual disturbances. Similar roles are evident among traditional healers in other ethnic groups [30–32], who also mediate between patients and spirits. Despite the widespread availability of modern medical treatments, these traditional practices persist, demonstrating the resilience of the Phu Thai community [29].

The Phu Thai people have maintained a profound connection with wildlife since ancient times, as their villages were traditionally surrounded by habitats rich in various wild animal species. Hunting has been a way of life for them, influenced by historical contexts such as inadequate public infrastructure and poverty. While modernization and regulations have curtailed hunting practices, some community members still hunt porcupines and deer for specific ceremonies during the third lunar month and the rice-planting season to honor ancestral traditions [33]. These practices promote community cooperation and reinforce family networks [33]. Similar observations were made by Heering [34] and Pennington [35], who found that the Hmong, Lahu, and Akha communities in Thailand also continue hunting to uphold ancestral traditions and strengthen communal ties.

During the COVID-19 pandemic, the Phu Thai community demonstrated strong self-protection behaviors and awareness, influenced by information from television, telecommunication, government agencies, and community leaders. They took proactive measures, such as planting fast-growing crops to ensure food security, practicing social distancing, wearing masks, and avoiding traditional ceremonies during lockdowns. The community collaborated closely with public health officials and local leaders in monitoring and supporting infected individuals, showcasing a solid commitment to epidemic self-protection.

Although the COVID-19 news is linked to the consumption of wild animals, the Phu Thai people believe that local wild animals are clean because they live in forests and eat fruit. During ancestor respect seasons, they prioritize buying and cooking wild animals in ancestral styles, believing it brings happiness to their ancestors. Little self-protection was seen in butchering practices, with no gloves worn and cleaning done during food preparation. At present, some Phu Thai people still consume raw meat and blood, such as wild boars and rabbits. As a result, the study’s findings highlight the need to change community engagement practices stemming from beliefs about resource scarcity and attitudes about zoonotic disease prevention. The adverse impact of spirituality on cultural domains, such as food consumption and nutrition, must be addressed and highlighted by villagers through appropriate intervention for risk communication [36, 37].

During the COVID-19 pandemic, the Phu Thai ethnic group hired “Mor Yaow” to perform the ceremony online, over the telephone, or through video call programs such as Line or Zoom Meeting Platform. Adjustments were made, such as reducing the number of “Mor Yaow” and musicians and using background music instead of live music for convenience and to suit the needs of those far away. They adhered to mask-wearing and social distancing principles, strictly following the planned ceremony sequence of their culture, showcasing their steadfastness in their cultural practices [38]. The Phu Thai has viewed illness as a divine punishment and attributed this belief to why conventional therapies have not been effective [36]. This perception underscores the cultural imposition, which refers to healthcare workers potentially imposing their systems and values [37].

## CONCLUSION

The study results highlighted the complex relationship between cultural practices, health behaviors, and wildlife interactions among the Phu Thai ethnic group in Nong Sung District, Mukdahan Province, Thailand. The Phu Thai demonstrate resilience and cultural pride by preserving their ancestral traditions and adapting to modern health challenges. However, practices such as wildlife consumption and traditional healthcare beliefs underscore the importance of culturally tailored public health interventions to mitigate zoonotic disease risks and improve overall health literacy.

The findings emphasize the need for a holistic approach, integrating local knowledge and traditions into health education and disease prevention strategies by organizing discussions among key stakeholders, including community members, leaders, traditional healers, public health officers, and local authorities, to gather feedback and develop effective policies and interventions. Strengthen healthcare accessibility, promote safe wildlife interactions, and foster community engagement to achieve sustainable health outcomes. In addition, incorporating OH principles can enhance collaboration between the public health sectors and local communities, thereby supporting the prevention of zoonotic diseases while respecting cultural diversity.

The study’s limitation was the small sample size, potentially affecting the generalizability of the findings. However, our sample sizes were saturated according to the guidelines of the qualitative research methodology [25]. In addition, we used triangulation analysis approaches by gathering data from multiple sources (IDIs, KIIs, and FGDs) to cross-check and validate the findings with the respondents to minimize potential biases. We also carefully selected respondents from various backgrounds within the Phu Thai community to ensure a diverse range of viewpoints. Despite these limitations, the findings highlight the importance of culturally sensitive and community-specific approaches to health education and disease prevention strategies and interventions.

## RECOMMENDATIONS

Based on the results, we recommend the promotion of safe wildlife interaction and consumption, enhancing health education, and implementing regular health screenings to prevent disease spread while respecting traditions. To implement these recommendations effectively, government and local agencies should prioritize the development and implementation of community-based interventions for advocating health education on zoonotic diseases among the Phu Thai people. First, there should be discussions among the Phu Thai community members, community leaders, traditional healers, public health officers, local government, and relevant stakeholders to gain feedback. This feedback can be used to develop and improve policies and regulations that support safer wildlife interactions, enhance disease prevention, and thus reduce the risk of zoonotic diseases among the Phu Thai ethnic group. In addition, community members should be informed of health risks by key community actors, including local community leaders, VHVs, animal health workers, traditional healers, public health officers, and relevant stakeholders. By combining these strategies with culturally sensitive education and community engagement, the Phu Thai community can reduce disease risks while preserving traditional practices.

To investigate cultural and health practices associated with wildlife interactions and identify zoonotic disease transmission risks, future research should involve a diverse range of respondents from Phu Thai and other ethnic groups across a variety of ages and professions. Understanding these risks can facilitate the development of targeted and effective interventions for specific high-risk groups, promoting health-conscious behaviors, respecting diverse cultural backgrounds, and promoting cultural diversity, sustainability, and overall well-being [7, 39–41].

Moreover, future research should extend to other ethnic groups and explore broader cultural contexts to develop more inclusive interventions. This study can serve as a foundation for culturally sensitive policies that honor traditional practices while prioritizing public health and environmental sustainability. Furthermore, we recommend adopting the OH strategy to contribute to achieving SDGs 3 (health and well-being) and 12 (responsible consumption and production) [42, 43].

## AUTHORS’ CONTRIBUTIONS

KS1, NB, KT, and TP: Designed the study. KS1, NB, and KT: Wrote the manuscript. KS1, NB, KT, TP, WT, PB, PK, WC, CS, and KS2: Collected, analyzed, and interpreted the data. KS1 and NB: Critically reviewed and edited the manuscript. All authors have read and approved the final manuscript.
